# Enhanced Structural Decoupling and Spatiotemporal Evolution of Thermal–Mass Coupling in LaNi_5_-Based Solid-State Hydrogen Storage Reactors

**DOI:** 10.3390/ma19071308

**Published:** 2026-03-26

**Authors:** Tao Wu, Yayi Wang, Yuhang Liu, Yong Gao, Rengen Ding, Jian Miao

**Affiliations:** 1 Institute of New Energy Science and Technology, Xi’an Technological University, Xi’an 710021, China; wt992312126@163.com (T.W.); wangyayi0219@163.com (Y.W.); 2 School of Materials and Chemical Engineering, Xi’an Technological University, Xi’an 710021, China; 18392734583@163.com; 3 College of Chemistry and Chemical Engineering, Inner Mongolia University, Hohhot 010021, China

**Keywords:** metal hydrides, LaNi_5_, thermal–mass coupling, spatiotemporal evolution, structural decoupling

## Abstract

Hydrogen energy is pivotal to the global energy transition, and the development of high-efficiency, safe hydrogen storage technologies constitutes a prerequisite for its large-scale commercialization. Kinetic bottlenecks including slow reactions, delayed front propagation, and marked spatial heterogeneity driven by strong thermal–mass transfer coupling restrict the engineering application of solid-state metal hydrides. However, the current research mainly focusing on overall performance lacks a systematic understanding of the spatiotemporal evolution mechanisms and their intrinsic links to internal structural control. In this work, a 3D multiphysics model of a LaNi_5_-based reactor is developed to systematically elucidate spatiotemporal evolution patterns, facilitating the proposal of a structural decoupling framework based on synergistic thermal–mass resistance reconfiguration. Both absorption and desorption show distinct three-stage evolution, shifting from kinetic dominance to transfer limitation: absorption causes core self-inhibition via heat-hydrogen supply mismatch, leading to much lower core than surface storage capacity; desorption results in significant inner-layer lag due to endothermic cooling-driven pressure drops. Thermal–mass coupling-induced inverted spatiotemporal evolution is identified as the root cause of spatial heterogeneity. Quantitative comparison of straight-pipe, spiral-tube, and honeycomb structures reveals that internal architectures achieve effective thermal–mass decoupling through expanded heat-exchange areas, reconstructed diffusion pathways, and optimized heat source distribution. Notably, the honeycomb structure with a parallel micro-unit network achieves 89.1% and 86.6% reductions in absorption and desorption times, respectively, showing superior dynamic performance and field uniformity. This study provides a theoretical basis for the mechanism-driven design and synergistic performance optimization of high-efficiency solid-state hydrogen storage reactors.

## 1. Introduction

Solid-state hydrogen storage has emerged as a core research direction due to its high volumetric storage density, mild operating conditions, and intrinsic safety [1,2]. As a key component of this technology, the solid-state hydrogen storage reactor determines the overall kinetic performance and hydrogen sorption rates through its heat and mass transfer capabilities [3,4,5]. Thus, in-depth investigation into the heat and mass transfer behaviors and their coupling characteristics during hydrogen absorption and desorption is imperative.

Insufficient heat transfer elevates equilibrium pressure, reduces thermodynamic driving force, and decelerates reaction kinetics, while simultaneously inducing significant temperature gradients and thermal hysteresis that prolong overall reaction duration [6,7]. For mass transfer, hydrogen sorption proceeds through three sequential stages—gas-phase diffusion, surface adsorption, and lattice diffusion—with impediments in any stage causing local concentration inhomogeneities, diminished driving force, and delayed reaction completion [8]. Heat and mass transfer are strongly spatiotemporally coupled: elevated temperatures increase equilibrium pressure and attenuate hydriding driving force, whereas concentration gradient fluctuations alter reaction heat generation rates, further exacerbating temperature field non-uniformity [9,10]. This interplay yields distinct stage-wise evolution and pronounced spatial heterogeneity during the reaction [11], thus identifying thermal–mass coupling as the core mechanism of reactor kinetic lag and efficiency loss [7,12]. This limitation can be further interpreted as a spatiotemporal mismatch between reaction heat release or supplement during hydrogen sorption and the comprehensive heat–mass transport capacity of the bed. To mitigate these coupling-induced limitations, structural optimization has become an effective strategy for enhancing heat and mass transfer performance. Most of these strategies are primarily developed from the perspective of thermal resistance reduction or heat-transfer-area expansion, with performance evaluation mainly based on global temperature control or reaction time. By integrating internal or external heat-exchange configurations [13,14], researchers can shorten heat conduction pathways [15], expand effective heat-transfer areas [16], or perturb the hydrogen flow field to redistribute heat flux [11,17], thereby facilitating rapid hydrogen diffusion and reaction kinetics and ultimately achieving synergistic thermal–mass transfer enhancement.

Extensive studies have demonstrated that the core challenge impeding the practical application of solid-state hydrogen storage reactors (metal hydride beds, MHBs) stems from the inherent limitations and strong spatiotemporal coupling of heat and mass transfer processes [18]. Specifically, the low effective thermal conductivity of hydride beds induces significant thermal diffusion lag, while the pore structure, permeability, and flow field characteristics of the reactor restrict gas-phase hydrogen replenishment and govern the spatial distribution of hydrogen concentration gradients. The mutual interplay between temperature and concentration fields further gives rise to non-uniform reaction kinetics throughout the reactor [19,20]. Nakagawa et al. [21] established a two-dimensional heat and mass transfer model for LaNi_5_ alloy beds, verifying that the low thermal conductivity of the alloy matrix constitutes the primary bottleneck limiting the overall reaction rate. B.S. Sekhar et al. [22] systematically evaluated reactor performance under diverse heat exchanger configurations, confirming heat transfer limitation as the dominant factor controlling reaction kinetics and proposing the expansion of heat transfer area as a highly efficient mitigation approach. X.S. Bai et al. [23] integrated bionic tree-like fins into MH reactors to enhance heat transfer efficiency, validating that the rational design and optimization of internal components represent a feasible and effective technical strategy. A. H. Eisapour et al. [24] conducted in-depth investigations on helical tube configurations, concluding that helical structures exhibit superior heat transfer performance and thus enable elevated hydrogen storage rates.

In terms of mass transfer limitations, Mat & Kaplan et al. [25] pointed out that regions adjacent to the reactor wall and the outer layers of hydride beds undergo preferential reaction owing to sufficient gas-phase hydrogen supply, whereas distinct reaction fronts form in the internal regions due to mass transfer delay. Wu et al. [26] adopted helical tube structures to induce radial convective circulation, thereby accelerating the dynamic replenishment of hydrogen in the reactor. Notably, heat and mass transfer processes are not mutually independent but exhibit strong spatiotemporal coupling effects. Previous review studies [2,10] elucidated this coupling mechanism, revealing that the mismatch between heat and mass transfer rates leads to pronounced spatiotemporal non-uniformity in the reactor. This phenomenon is specifically characterized by the temporal asynchrony between peak temperature and peak hydrogen sorption rate, which identifies the decoupling of thermal diffusion and species transport as the intrinsic cause of kinetic inhibition in solid-state hydrogen storage reactors. Although significant progress has been made in the heat and mass transfer and thermal–mass coupling of solid-state hydrogen storage reactor, existing research primarily focuses on the quantitative evaluation of reaction rates or overall hydrogen sorption performance. A systematic characterization of the temporal evolution patterns and axial–radial spatial heterogeneity of thermal–mass coupling behaviors during sorption processes remains insufficient. Specifically, there is a lack of unified mechanistic understanding and quantitative analysis regarding how the temporal mismatch between reaction heat effects, dynamic variations in equilibrium pressure, bed thermal diffusion, and gas replenishment capability jointly shapes the reaction front propagation and the evolution of temperature and hydrogen storage fields. On the other hand, optimization studies of internal heat-exchange configurations often aim at increasing the equivalent heat-transfer area or enhancing overall thermal performance. Evaluation metrics are predominantly focused on sorption time or average reaction rates, with insufficient attention paid to the mechanisms by which different structures spatially regulate heat source distribution, reaction front morphology, and spatial non-uniformity. Furthermore, systematic comparative evaluations of thermal–mass coupling evolution under standardized models and operating conditions remain sparsely documented. Therefore, it is imperative to systematically investigate the thermal–mass transfer characteristics, coupled reaction behaviors, and structural optimization models to achieve the safe and efficient design of MH reactors and meet the practical application requirements of solid-state hydrogen storage.

It should be noted that the “structural decoupling enhancement design framework” proposed in this study distinguishes itself from conventional “thermal resistance network optimization” in terms of both research objectives and design logic. The existing research on structural enhancement predominantly targets the reduction in global thermal resistance or the expansion of heat exchange areas, with evaluation metrics typically focusing on average temperature levels or the reduction in reaction time, thereby emphasizing the intensification of a single heat transfer path. In contrast, this work performs structural reconfiguration based on the spatiotemporal evolution mechanism of thermal–mass coupling, specifically addressing the dynamic mismatch between reaction thermal effects during hydrogen absorption and desorption and the hydrogen transport process. The key lies not merely in increasing thermal conductivity, but in simultaneously compressing the characteristic path scales of both heat and mass transfer through internal configuration reconstruction. This enables coordinated dynamic responses of the temperature and concentration fields and mitigates the coupled inhibition effect of localized heat accumulation on reaction front propagation and gas replenishment. From a methodological perspective, this approach represents a structural design framework oriented toward multi-physical field synergistic response regulation, rather than a single-objective thermal resistance optimization strategy.

Accordingly, herein, we focus on the thermo-mass transfer coupling behaviors of LaNi_5_-based hydrogen storage reactors during hydrogen sorption/desorption cycles. And the systematic analysis of the temporal evolution laws of temperature fields and hydrogen storage capacity distributions, as well as the axial–radial spatial distribution patterns within the reactor are investigated systematically. Building upon this analysis, a comparative investigation is carried out to clarify the regulatory mechanisms of three types of internal structural configurations consisting of (i) straight tubes, (ii) spiral tubes, and (iii) honeycomb tubes on the thermo-mass coupling effects and reaction kinetic properties. Collectively, this work lays a theoretical foundation and provides quantitative data support for the safe, high-efficiency structural optimization design of solid-state hydrogen storage reactors.

## 2. Materials and Methodologies

### 2.1. Numerical Simulation Method

**Figure 1:**
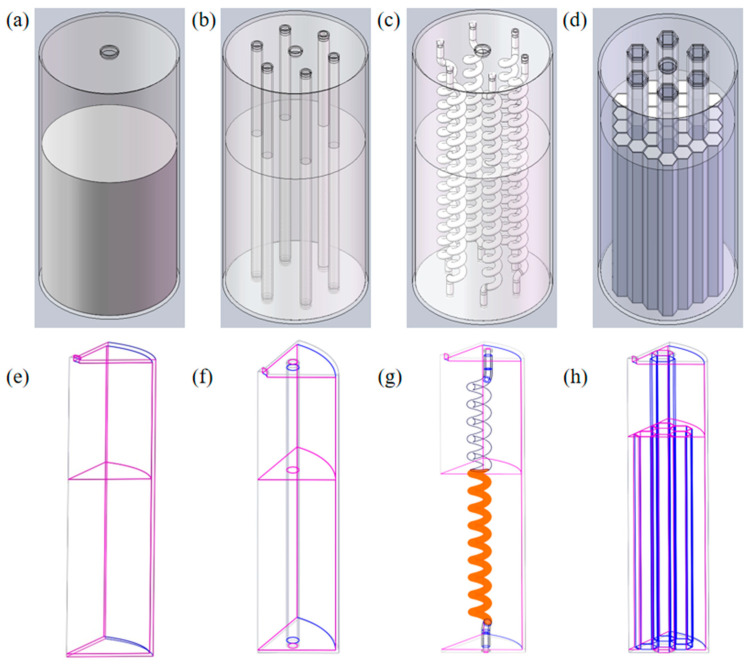
Schematic diagrams of the basic structure and various internal heat-exchange structures of the solid-state hydrogen storage reactor, along with their corresponding one-sixth circumferential symmetric computational domains: (**a**) basic structure without internal heat-exchange components; (**b**) straight pipe structure; (**c**) spiral tube structure; (**d**) honeycomb structure; (**e**) one-sixth computational domain of the basic structure; (**f**) one-sixth computational domain of the straight pipe structure; (**g**) one-sixth computational domain of the spiral tube structure; (**h**) one-sixth computational domain of the honeycomb structure.

Structure and Parameters. The solid-state hydrogen storage reactor used in the simulation, based on LaNi_5_ alloy, is designated as the basic structure. It is constructed primarily from 316L stainless steel with a cylindrical overall geometry. The parameters of the baseline structure are as follows: height H = 102 mm, radius R = 26 mm, hydrogen inlet/outlet diameter D = 4 mm, and wall thickness L = 1 mm. The hydride alloy is filled to a radius r = 25 mm and a height h = 60 mm. The remaining unfilled volume within the reactor serves as reserved space for the expansion of the alloy during hydrogen absorption. Various internal heat-exchange configurations are implemented by incorporating specific components into the baseline reactor. The straight pipe structure consists of six axial heat-exchange channels uniformly distributed within the reactor, where the tube walls are in direct contact with the hydride. These tubes have an outer diameter (OD) of 4 mm and an inner diameter (ID) of 3 mm. The spiral tube structure replaces the straight pipes with six helical heat-exchange channels featuring a pitch of 6 mm and 14 coils. The helical radius is 2.5 mm, with a tube OD of 2.5 mm and an ID of 1.5 mm. The honeycomb structure involves constructing honeycomb support cells within the hydride filling region, with the alloy filled both inside and outside the cells to ensure uniform distribution. The inscribed circle diameter of each honeycomb cell is 6 mm with a wall thickness of 1 mm. Additionally, six hexagonal heat-exchange pipes, also with an inscribed circle diameter of 6 mm, are arranged uniformly along the axial direction (see Figure 1). 

The selection criteria for these internal configurations are based on a stepped evolution logic regarding the characteristic scales and pathways of thermal and mass transport. To ensure full reproducibility of the numerical model, the detailed geometric dimensions and boundary parameters for all reactor configurations are summarized in Table 1.

The straight pipe structure aims to evaluate the enhancement efficiency of a single axial, low-resistance channel at the macroscale. The spiral tube structure, by introducing geometric curvature effects, explores the potential for performance improvement through heat-transfer area expansion and potential gas-phase disturbances. In contrast, the honeycomb structure represents a transition from macroscale bulk transmission toward distributed micro-unit local equilibrium control. A comparative analysis of these structures with distinct physical characteristic lengths enables a deep investigation into the synergistic enhancement mechanisms of spatial architectural features regarding heat source reconfiguration and transport path shortening. All reactor configurations share identical boundary conditions, with water serving as the heat-transfer fluid (HTF) at a flow velocity of 1 m/s. The thermo-physical parameters utilized in the simulation are summarized in Table 2.

Due to the variations in the volume of internal heat-exchange configurations and the 25% maximum expansion rate of LaNi_5_, the filling height of the hydride alloy within the reactor was adjusted for each configuration to ensure a constant total alloy volume. The specific filling heights for the different heat-exchange configurations are summarized in Table 3.

**Figure 2:**
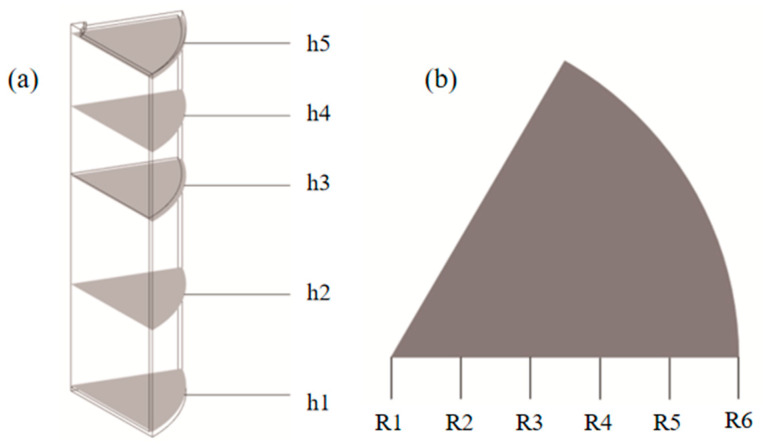
Schematic diagram for selecting axial and radial characteristic positions (**a**) h1–h5 represent axial cross-sections, (**b**) R1–R6 denote radial monitoring points.

Five key cross-sections of the reactor are selected to facilitate the analysis of internal reaction patterns. The heights of these sections are defined as follows: h1 = 0 (bottom surface of the MHB), h2 = 30 mm (middle layer of the MHB), h3 = 60 mm (top surface of the MHB), h4 = 80 mm (middle layer of the reserved expansion space), h5 = 100 mm (top surface of the expansion space within the reactor). To perform a more detailed analysis of the internal dynamic response, six radial monitoring points are selected on the h2 = 30 mm plane at radii of R1 = 0, R2 = 5 mm, R3 = 10 mm, R4 = 15 mm, R5 = 20 mm, and R6 = 25 mm. Among these, R1 is located on the central axis of the reactor and R6 is at the contact point between the hydride alloy and the reactor wall. The sections h1 to h5 and points R1 to R6 are utilized to analyze the axial and radial distribution patterns, as shown in Figure 2.

Because the variations in the dimensions of different heat-exchange structures result in inconsistent filling heights of the hydride alloy, the specific heights of the selected cross-sections for the basic structure, straight pipe structure, spiral tube structure, and honeycomb structure are determined according to the aforementioned rules and are detailed in Table 4.

### 2.2. Mathematical Model

The hydrogen sorption process within the hydride alloy of the reactor involves coupled heat and mass transfer. The following governing equations are employed in the numerical simulation.

#### 2.2.1. Mass Conservation Equation

For the gas-phase hydrogen, given a constant porosity, the mass conservation equation is expressed in Equation (1).

(1) $$\frac{\partial }{\partial t}\left(\varepsilon \rho_{g}\right)+\nabla \cdot \left(\rho_{g}\vec{u}\right)=-S_{m}$$

where *ε* is the porosity of the hydride alloy, $\rho_{g}$ is the hydrogen density, kg/m^3^, $S_{m}$ is the mass source term, kg/(m^3^·s), and $\vec{u}$ is the hydrogen velocity vector, m/s.

The hydrogen density, which follows the ideal gas equation of state, is defined in Equation (2).

(2) $$\rho_{g}=\frac{P_{g}M_{g}}{RT}$$

where $P_{g}$ is the hydrogen pressure, Pa, $M_{g}$ is the molar mass of hydrogen, R is the universal gas constant, J/(mol·K), and T is the temperature, K.

During the hydrogen sorption process of metal hydrides, the mass variation in the hydride bed is equal and opposite to that of the gas-phase hydrogen. Thus, the mass conservation equation for the solid phase can be expressed in Equation (3).

(3) $$(1-\varepsilon )\frac{\partial \rho_{s}}{\partial t}=S_{m}$$

$\rho_{s}$ represents the mass density of the hydride alloy per unit volume (unit, kg/m^3^).

Hydrogen absorption occurs when the hydrogen pressure $P_{g}$ within the hydride alloy exceeds the equilibrium pressure for the absorption process $P_{\mathrm{eq}\_a}$. Conversely, hydrogen desorption is triggered when $P_{g}$ is lower than the desorption equilibrium pressure $P_{\mathrm{eq}\_d}$.

(4) $$S_{m}=C\exp\left(-\frac{E}{RT}\right)\left|\frac{P_{g}-P_{\mathrm{eq}}}{P_{\mathrm{eq}}}\right|\cdot (\rho^{*}-\rho_{s})\cdot \mathrm{sgn}(P_{g}-P_{\mathrm{eq}})$$

Assuming the volume of the MHB remains constant, only the increase or decrease in alloy density during the reaction is taken into account. To provide a unified mathematical description for both absorption and desorption states [29], the mass source term is expressed in Equation (4). The parameter values used for both hydrogen absorption and desorption are listed in Table 5.

In the equation, denotes the reaction activation energy, kJ/mol; represents the saturated density of the hydride alloy at its maximum hydrogen capacity, while is the density of the alloy in its hydrogen-free state, kg/m^3^; is the reaction rate constant, s^−1^; represents the instantaneous density of the metal hydride during the sorption process, kg/m^3^; and is the equilibrium pressure, Pa.

The equilibrium pressure can be determined using the Van’t Hoff equation [30]. During the hydrogen sorption process, temperature is the dominant factor influencing the equilibrium pressure [31], as expressed in Equation (5).

(5) $$\ln\left(\frac{p_{\mathrm{eq}}}{p_{\mathrm{ref}}}\right)=A-\frac{B}{T}$$

In the equation, $P_{\mathrm{ref}}$ denotes the reference pressure during the hydrogen sorption process. The constants A and B are the reaction coefficients for the hydride alloy under reaction and equilibrium conditions, which are determined through data fitting [32,33,34]. Their values used in the simulation are listed in Table 6.

#### 2.2.2. Energy Conservation Equation

To calculate the temperature distribution within the hydrogen storage bed, an energy conservation equation is established based on the assumption that Local Thermal Equilibrium (LTE) is reached between the gas phase and the reaction bed. The corresponding governing equation is expressed in Equation (6):

(6) $$(\bar{\rho C_{p}})\frac{dT}{dt}+\nabla \cdot \left(\rho_{g}C_{p,g}\vec{u}T\right)=\nabla \cdot \left(\lambda_{\mathrm{eff}}\nabla T\right)+S_{T}$$

where $C_{p,g}$ is the specific heat capacity of hydrogen gas (unit, J/(kg·K)), $\bar{\rho C_{p}}$ represents the effective volumetric heat capacity of the bed (unit, J/(m^3^·K)), $\lambda_{\mathrm{eff}}$ is the effective thermal conductivity of the metal hydride bed (unit, W/(m·K)), and ${\vec{S}}_{T}$ is the energy source term in the energy equation (unit, W/m^3^).

The hydride alloy is modeled as a porous structure with fluid–solid coupling. Based on the volume-weighted method, $\bar{\rho C_{p}}$ is expressed in Equation (7), while $\lambda_{\mathrm{eff}}$ is treated as a constant to represent the global heat transfer capability of the bed, accounting for the contact resistance between particles.

(7) $$\bar{\rho C_{p}}=(1-\varepsilon )\rho_{s}C_{p,s}+\varepsilon \rho_{g}C_{p,g}$$

The subscripts s and g represent the solid and gas phases, respectively. $C_{p,s}$ is the specific heat capacity of the hydride alloy (unit, J/(kg·K). The energy source term ${\vec{S}}_{T}$ describes the heat exchange during the hydrogen sorption process, which can be calculated based on the hydrogen mass transfer rate and the reaction enthalpy change ΔH in Equation (8).

(8) $$S_{T}=S_{m}\left[\Delta H-T\left(C_{p,g}-C_{p,s}\right)\right]$$

where ΔH is the enthalpy of reaction.

#### 2.2.3. Momentum Conservation Equation

The momentum conservation equation involved in the reaction is expressed in Equation (9):

(9) $$\frac{1}{\varepsilon }\left[\frac{d\rho_{g}}{dt}\vec{u}+\frac{1}{\varepsilon }\nabla \left(\rho_{g}\vec{u}\cdot \vec{u}\right)\right]=-\nabla p+\nabla \cdot \tau +\rho_{g}\vec{g}+{\vec{S}}_{u}$$

where ${\vec{S}}_{u}$ is the momentum source term (unit, N/m^3^). Since the metal hydride powder within the MHB is treated as a homogeneous porous medium, the internal velocity field $\vec{u}$ follows Darcy’s Law, which is defined in Equation (10).

(10) $${\vec{S}}_{u}=-\left(\frac{\mu }{K}\right)\vec{u}$$

where μ denotes the dynamic viscosity (unit, Pa·s). K represents the permeability (unit, m^2^). During the simulation, the initial and boundary conditions are prescribed as follows. The hydride alloy is defined as a porous medium, the ambient environment of the reactor is set as an isothermal air field, and the heat-transfer fluid (HTF) within the channels is water. This configuration aims to simplify the computational model and prevent precision degradation caused by external environmental disturbances. This approach improves overall computational efficiency and allows the research to focus on the internal heat and mass transfer mechanisms and the analysis of structural optimization for the reactor.

For the numerical simulation, it is assumed that the model is in a state of thermodynamic equilibrium under initial conditions. The initial conditions are defined in Equation (11).

(11) $$T_{0}=T_{\mathrm{ev}}=T_{\mathrm{in}}=T_{\mathrm{out}}=T_{w}=T_{s}=T_{g}$$

$T_{0}$ is the initial temperature, $T_{\mathrm{ev}}$ denotes the ambient temperature, $T_{\mathrm{in}}$ and $T_{\mathrm{out}}$ represent the inlet and outlet temperatures of the fluid, respectively, $T_{w}$ is the temperature of the water medium, $T_{s}$ and $T_{g}$ are the temperatures of the hydride alloy and hydrogen, respectively.

(12) $$v_{g}=v_{w}=0$$

where $v_{g}$ is the hydrogen velocity and $v_{w}$ is the velocity of the HTF, m/s.

For the hydrogen absorption reaction, the initial conditions for the hydride alloy density and hydrogen pressure are in Equation (13).

(13) $$\rho_{s}=\rho_{\mathrm{emp}},\;P=P_{\mathrm{eq}\_a}$$

For the hydrogen desorption reaction, the initial conditions for the density and pressure are in Equation (14):

(14) $$\rho_{s}=\rho_{\mathrm{sat}},\;P=P_{\mathrm{eq}\_d}$$

For reactors with different heat-exchange structures, typical sorption temperatures for LaNi_5_ are selected. During absorption, the initial alloy temperature $T_{s}$ and ambient temperature $T_{\mathrm{ev}}$ are both set to 293 K, with a hydrogen inlet pressure of 1 MPa. For desorption, the initial alloy temperature $T_{s}$ and ambient temperature $T_{\mathrm{ev}}$ are set to 313 K, with a hydrogen outlet pressure of 0.1 MPa. During the hydrogen sorption reactions, the heat-transfer fluid temperature $T_{w}$ equal to $T_{s}$, and the fluid velocity $v_{w}$ is maintained at 1 m/s. The heat exchange between the hydride alloy region and the external environment is governed by the following boundary condition Equation (15).

(15) $$-\lambda_{\mathrm{eff}}\frac{\partial T}{\partial \vec{n}}=h(T-T_{\mathrm{ev}})$$

h is the convective heat transfer coefficient (unit, W/(m^2^·K)).

The convective heat transfer coefficient is applied only to the external shell surface of the reactor to approximate the constant-temperature water bath environment used in the reference experiment. For the internal heat exchange tube walls, the energy exchange between the fluid and solid domains is solved using the conjugate heat transfer (CHT) approach.

To investigate the influence of different heat-exchange structures on the heat and mass transfer of the reactor, the computational domain for the heat transfer analysis is defined to encompass both the hydride alloy and the internal heat-exchange structures. Conversely, the mass transfer analysis exclusively focuses on the diffusion and reaction processes within the hydride alloy. The hydrogen sorption process in metal hydrides involves gas flow, chemical reactions, and the associated release or absorption of heat. To facilitate mechanistic analysis and enhance computational efficiency, the physical processes involved in hydrogen sorption are simplified based on the following assumptions: (i) hydrogen behaves as an ideal gas following the ideal gas equation of state. (ii) The thermo-physical properties of the hydride alloy and hydrogen remain constant during the reaction process. (iii) The metal hydride is a uniformly filled and isotropic porous medium within the reactor. (iv) The temperature and flow velocity of the heat-transfer fluid remain constant. (v) Local Thermal Equilibrium (LTE) is maintained between the gas phase and the metal hydride during the sorption process. (vi) The thermo-physical parameters of the metal hydride, such as porosity, remain constant throughout the sorption process. (vii) Volumetric changes due to the expansion and contraction of the metal hydride during sorption are neglected.

### 2.3. Numerical Solution and Validation

Numerical simulations are performed using ANSYS Fluent 2024 R1 software to solve the conservation equations for mass, energy, and momentum based on the Finite Volume Method. The porous media module in Fluent is employed to simulate hydrogen flow within the LaNi_5_ hydride bed, while the standard k-ε turbulence model is utilized to describe the flow characteristics of the heat-transfer fluid. To ensure numerical consistency among different structural configurations, the standard k-ε turbulence model was uniformly adopted for the coolant flow calculations in this study. A sensitivity analysis was further conducted for the helical tube configuration by comparing the laminar and turbulence models. The results indicate that the predicted bed temperature evolution and hydrogen storage capacity curves are highly consistent. The temperature difference throughout the hydrogen absorption process is approximately 0.1 K, and the laminar model only shows a slightly delayed hydrogen absorption behavior. Accurate simulation of the LaNi_5_ reaction kinetics is achieved through User-Defined Functions (UDFs) written in C, which facilitate the dynamic coupling of the energy and mass source terms of the chemical reactions. Variable relationships are established by prescribing initial conditions, and source term calculation modules are constructed using the DEFINE_SOURCE macro to realize the coupled solution of mass and energy based on the governing equations. Finally, the UDF is integrated into the solver through interpretation and compilation to implement the loading and execution of reaction source terms.

**Figure 3:**
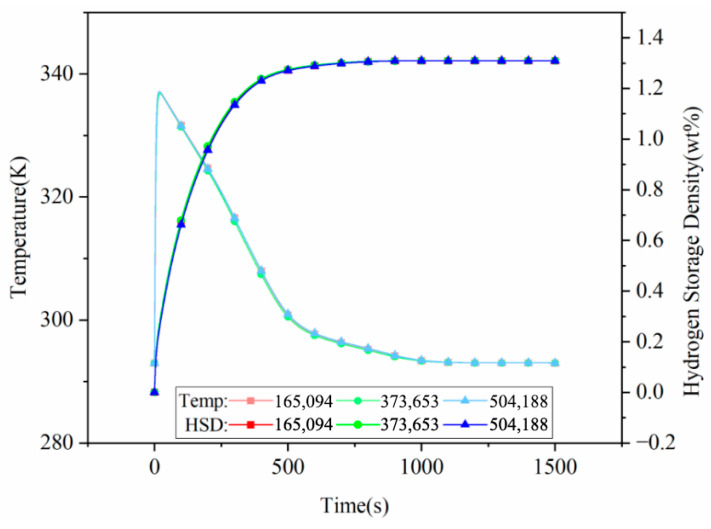
Comparison of hydrogen storage capacity and temperature over time for different mesh densities.

As shown in Figure 3, three mesh schemes with 165,094, 373,653, and 504,188 cells are developed. Under conditions of an alloy temperature of 293 K, an inlet pressure of 1 MPa, and an ambient temperature of 293 K, the temporal evolution of hydrogen storage capacity and temperature is compared across the different mesh densities. The results show that the curves for the three refinement levels are in good agreement. Consequently, the 165,094 mesh scheme is selected to optimize computational efficiency while ensuring sufficient accuracy.

**Figure 4:**
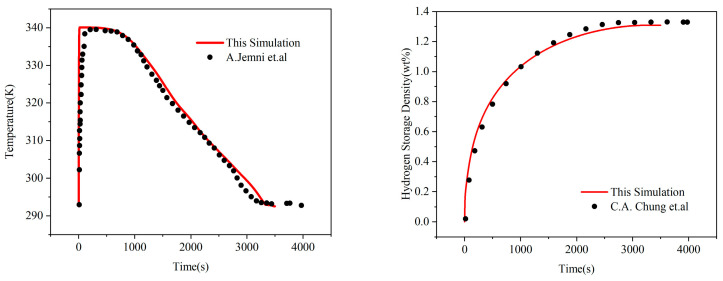
Model validation through the comparison of temperature and hydrogen storage capacity evolution between the simulation results and literature data from Jemni et al. [35] and Chung et al. [27].

In the numerical simulation of the MH reactor, as indicated in Equation (4), the calculation expressions for the mass source terms differ between the hydrogen absorption and desorption processes. Consequently, it is necessary to perform a validity check on the mathematical model of the reactor. This validation is conducted by comparing the temperature–time and hydrogen storage capacity–time curves. Since the parameters and operating conditions reported by Jemni et al. [35] and Chung et al. [27] are highly similar to those in this simulation, the simulation results are validated against their literature data. Specifically, the validations for both temperature and hydrogen storage capacity were performed under the absorption regime with a constant inlet pressure of 0.8 MPa and a constant cooling temperature of 293.15 K. The temperature evolution was monitored at a representative location of r=15 mm and z=35 mm, which corresponds to the point used by Chung et al. [27] and Point A in Jemni’s experimental setup [35]. The resulting temperature–time and hydrogen storage capacity–time curves during absorption show excellent agreement with the experimental data, as illustrated in Figure 4. The specific boundary conditions and thermophysical parameters utilized for this validation are summarized in Table 7. 

## 3. Results and Discussion

### 3.1. Heat–Mass Transfer Characteristics During H_2_ Absorption

Hydrogen adsorption in metal hydrides is essentially an intensely coupled process involving the interplay of heat transfer, mass transfer, and solid-phase reaction kinetics. Based on numerical simulation results (see Section 2.2 and Section 2.3), the thermal–mass transfer behaviors of LaNi_5_ beds during hydrogen absorption and desorption were analyzed systematically. It highlights the evolution patterns of temperature and hydrogen storage fields, reaction front propagation mechanisms, and critical rate-limiting links of the reaction. By probing into the spatiotemporal and structural coupling characteristics, this study provides a theoretical basis for designing and optimizing high-efficiency MH reactors. Hydrogen adsorption is inherently a strongly exothermic reaction restricted by heat and mass transfer rates, with its kinetics determined by the spatiotemporal matching between reaction heat release, bed heat dissipation, and mass transfer capacities. Elevated temperatures boost local equilibrium pressure, which suppresses the thermodynamic driving force and triggers a sustained thermal self-inhibition effect on the reaction. Concurrently, hydrogen transport into the metal hydride bed (MHB) is limited by diffusion pathways and permeation resistance, hindering reaction front propagation toward the core and leading to significant spatial heterogeneity. From the viewpoint of thermal–mass coupling imbalance, this section systematically analyzes the temporal evolution and spatial distribution of temperature and hydrogen storage fields, to reveal the spatiotemporal dynamic response mechanisms of the adsorption reaction under multi-physical field competition.

Objective transition points between the three stages are identified using the derivative of the average bed temperature (d*T*/d*t*) to ensure mechanistic rigor and reproducibility. The transition from Stage I to Stage II is defined as the moment when the temperature derivative approaches zero (dT/dt≈0). This point represents the instantaneous balance between the reaction heat generation rate and the system’s external heat dissipation rate, marking the shift from a rapid temperature surge to a plateau or gradual decline. The transition from Stage II to Stage III is identified as the entry into a low-speed thermal response interval. When ∣dT/dt∣ drops below the threshold of $10^{-2}\;K/s$, the intense coupled thermal response is considered to have substantially subsided, and the system transitions into a quasi-stable stage dominated by mass transfer limitations. The partitioning of the hydrogen storage capacity and reaction rate curves follows these temperature-derived characteristic time nodes to ensure physical consistency.

**Figure 5:**
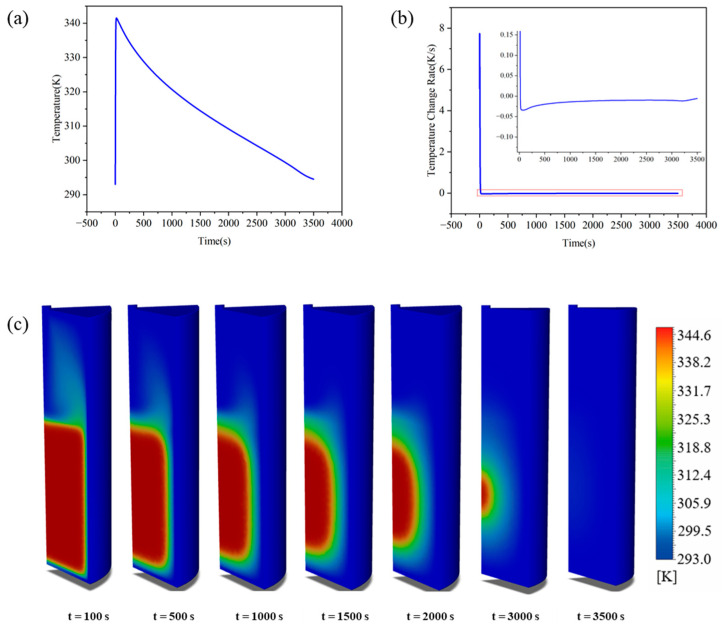
Temperature evolution in the hydride bed during absorption: (**a**) average bed temperature; (**b**) temperature change rate; (**c**) temperature distribution at different moments.

From the perspective of reaction control mechanisms, hydrogen adsorption in the MHB is not governed by reaction kinetics or diffusion alone, and it transitions temporally from reaction dominance to thermal-diffusion dominance. This shift stems from the spatiotemporal scale mismatch between reaction heat generation rate and bed thermal diffusion rate. Restricted by the bed’s thermal diffusion capacity and gas replenishment conditions, reaction heat cannot dissipate promptly, resulting in a three-stage temperature field evolution during absorption: (i) reaction-dominated rapid heating, (ii) competitive transition between heat release and thermal diffusion, and (iii) transfer-limited quasi-equilibrium (Figure 5a,b). Similar staged temperature trends have been reported by Jemni et al. [35] and Chung et al. [27], who observed distinct temperature peaks followed by non-linear decline in absorption processes. Building on these findings, this study further elucidates the physical mechanisms of these three consecutive stages. The initial stage (*t* < 20 s) is a reaction-dominated rapid heating phase driven entirely by intense exothermic heat, with the bed temperature surging to a transient peak of 341.5 K within 20 s. Given that the effective thermal conductivity of LaNi_5_ and most AB_5_-type alloys is typically below 3 W/(m·K), heat generation far exceeds thermal diffusion, causing heat accumulation and significant temperature gradients in the bed (Figure 5c), as widely documented in existing review articles [36,37]. More importantly, the rapid temperature rise elevates local equilibrium pressure, weakening the thermodynamic driving force markedly. This thermally induced self-inhibition effect dominates from the reaction onset and induces pronounced spatial heterogeneity, consistent with the numerical model of Hasnain et al. [19].

The intermediate stage (20 < *t* < 1000 s) corresponds to the transition phase where reaction heat release and thermal diffusion compete dynamically. The temperature peak of 341.5 K observed at 20 s denotes the dynamic equilibrium point at which the reaction heat generation rate is equivalent to the overall heat dissipation rate of the bed. As widely documented in existing literature [3,4,5], the reaction rate declines progressively under the inhibitory effect of elevated equilibrium pressure, which in turn allows heat dissipation to gradually take precedence and drives the system temperature downward. Around the 70 s mark, the discrepancy between the fading heat release from the reaction and the sustained heat dissipation capacity of the bed reaches its maximum magnitude; accordingly, the cooling rate peaks at 0.034 K/s before exhibiting a characteristic non-linear decay trend thereafter. The late stage (*t* > 1000 s) corresponds to the transfer-limited quasi-equilibrium phase, during which the cooling rate stabilizes at approximately 0.01 K/s and the system enters a prolonged slow recovery period—an observation evidenced by the profiles at *t* = 2000 s and *t* = 3000 s in Figure 1c. At this stage, the inner alloy layers sustain only a sluggish reaction, constrained by the elevated equilibrium pressure and extended lattice diffusion pathways. The heat released from this weak reaction is insufficient to induce measurable temperature fluctuations, and the residual heat is transferred to the outer wall exclusively via inefficient solid-phase conduction and limited gas-phase convection. Similar trends of late-stage temperature recovery and reaction attenuation have been reported in numerous investigations on metal hydride beds (MHBs) [38,39]. The physical origin of this behavior lies in the progressive depletion of the thermodynamic driving force under the dual constraints of temperature and pressure. This phased evolution not only verifies the competitive mechanism between heat and mass transfer but also establishes the essential thermodynamic framework for interpreting the kinetic inhibition of hydrogen storage capacity in subsequent stages.

**Figure 6:**
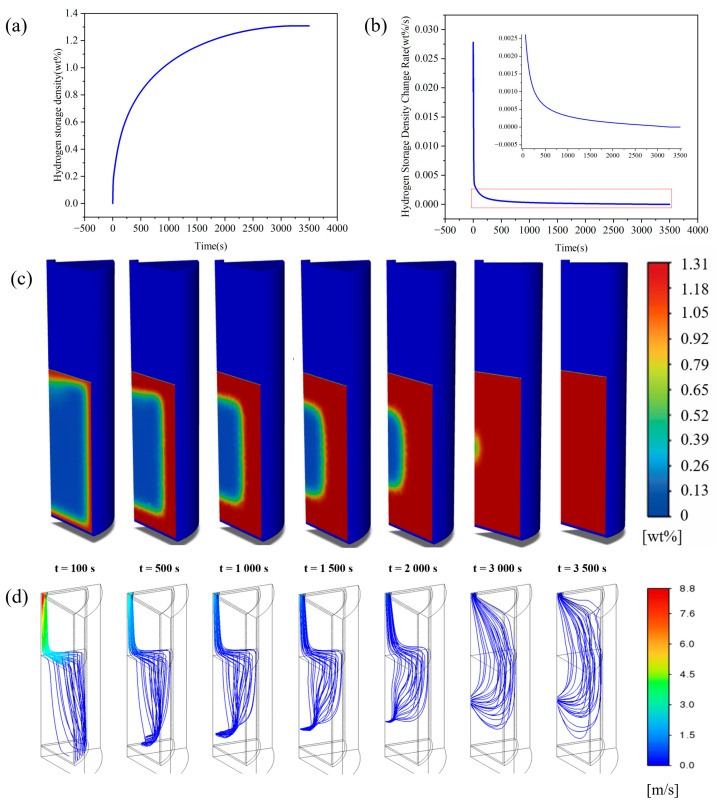
Hydrogen storage capacity and hydrogen flow field during the absorption process: (**a**) average bed hydrogen storage capacity; (**b**) hydrogen storage rate; (**c**) hydrogen storage distribution at different moments; (**d**) hydrogen flow field distribution at different moments.

Constrained by the spatiotemporal evolution of the temperature field elaborated above, the hydrogen storage capacity displays distinct staged kinetic characteristics that are closely correlated with the dynamic temperature variations over time. During the hydrogen absorption process, the storage capacity undergoes a three-stage evolutionary process: (i) a rapid surface adsorption stage, (ii) a deceleration stage governed by the synergistic effects of reaction heat release and hydrogen diffusion, and (iii) a quasi-equilibrium stage limited by heat–mass transport, as illustrated in Figure 6a,b. This typical staged kinetic behavior during hydrogen absorption has been extensively documented in previous investigations on metal hydride beds. Physically, this phenomenon reflects the transition of the rate-determining mechanism from surface reaction dominance to the dominance of thermal–mass transfer resistance [29].

The initial stage (t < 20 s) corresponds to the rapid adsorption phase, featured by a sharp ascent in hydrogen storage capacity. Driven by the high external hydrogen partial pressure, hydrogen molecules rapidly fill the porous structures and are preferentially adsorbed by the outer layers of the alloy matrix. Given the minimal gas-phase replenishment resistance near the reactor wall, hydrogen can swiftly migrate to the reaction interface through wall-adjacent transport pathways. A large quantity of active reaction sites are occupied within an extremely short timeframe, resulting in a steep upward slope in the hydrogen storage curve, as visualized at t = 100 s in Figure 6c,d. The middle stage (20 s < t < 1000 s) proceeds to the deceleration phase, which is governed by the synergistic effects of reaction heat accumulation and hydrogen diffusion. Although the hydrogen storage capacity sustains an increasing trend, its growth rate is thermally inhibited, declining sharply from approximately 0.02 wt%/s at the initiation to 0.0035 wt%/s at t = 20 s. As the reaction advances, the transport pathways extending to the inner alloy layers lengthen progressively, which elevates the diffusion resistance and thus induces a continuous reduction in the reaction rate. This observation aligns well with the existing findings that the elevation of equilibrium pressure induced by temperature rise and the intensification of diffusion resistance jointly contribute to the decay of reaction kinetics [40]. Distinct from most relevant studies that merely differentiate between the rapid adsorption and slow diffusion stages, the present work further identifies an intermediate non-linear stage regulated by the synchronous escalation of reaction thermal effects and gas replenishment resistance. The contour plots at t = 500 s and t = 1000 s in Figure 6c,d also demonstrate substantial discrepancies in the internal distribution of hydrogen storage capacity during this phase. Concurrently, the hydrogen flow velocity decreases from the initial value exceeding 8.8 m/s to below 3 m/s, which corroborates the deceleration characteristics of the reaction process under the coupled constraints of thermal effects and diffusion resistance. The late stage (t > 1000 s) refers to the transport-limited quasi-equilibrium phase, where the hydrogen storage capacity slowly approaches the plateau value and the storage rate gradually tends toward zero. This stage is designated as transport-limited on the grounds that the reaction rate is synergistically constrained by microscale lattice solid-phase diffusion and macroscale bed heat dissipation. A typical characteristic of this phase is the prolonged, slow trajectory toward equilibrium, which is regarded as an intrinsic attribute of the hydrogen absorption process in metal hydride systems [41]. On the one hand, restricted by the low thermal conductivity of the alloy, the residual internal heat cannot be dissipated rapidly; thermodynamic analysis indicates that the local equilibrium pressure thus remains at a relatively high level. On the other hand, the diffusion of hydrogen atoms within the metal lattice is extremely sluggish. This state of synergistic heat and mass transport limitation drives the hydride bed to gradually evolve into a quasi-equilibrium state, exhibiting a typical pattern of long-term slow tailing behavior. As the reaction proceeds, the asynchronous temporal.

**Figure 7:**
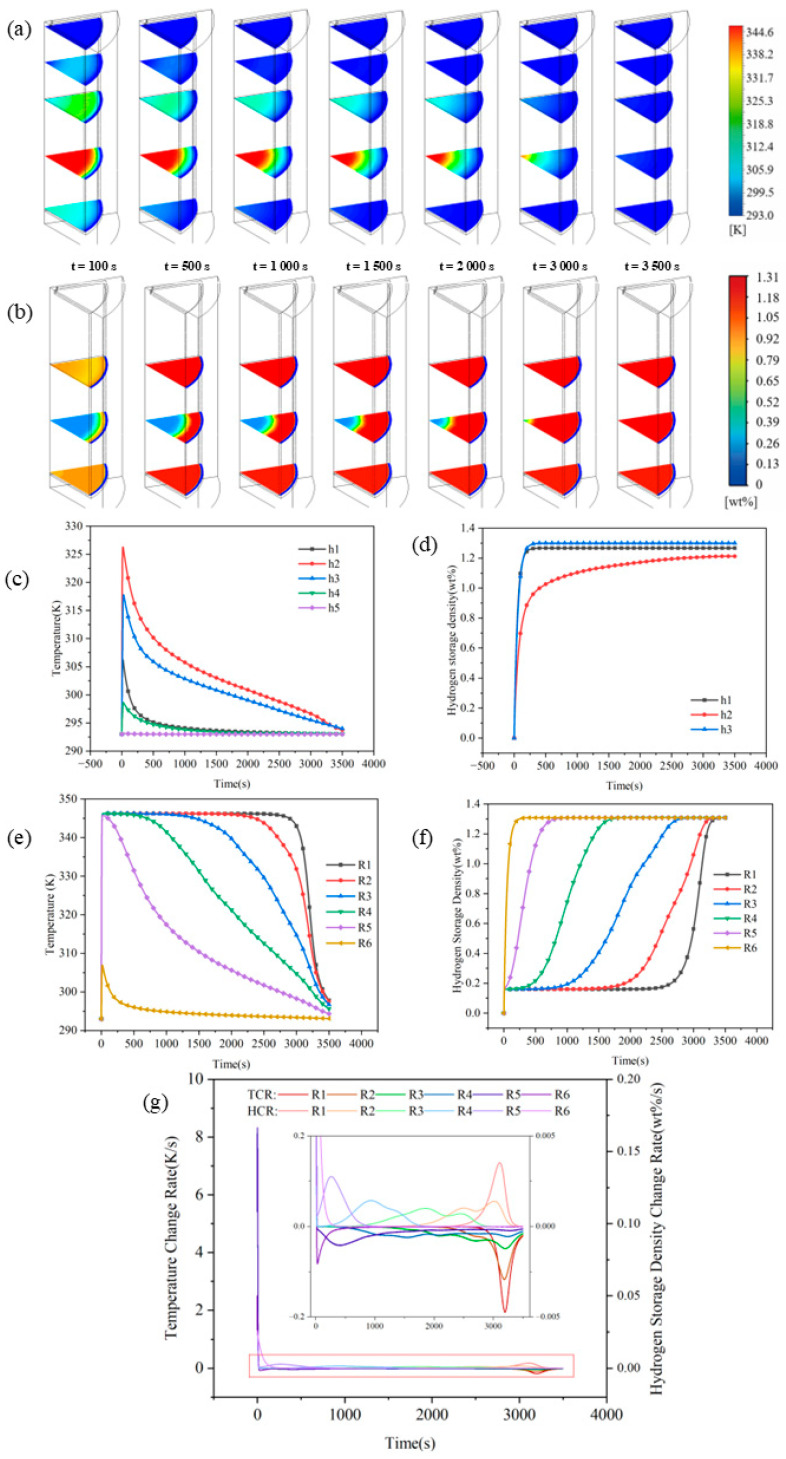
Axial and radial spatial evolution during absorption: (**a**) axial temperature distribution; (**b**) axial hydrogen storage capacity distribution; (**c**,**d**) temperature and hydrogen storage capacity at different cross-sections; (**e**–**g**) temperature, hydrogen storage capacity, and their rates of change at various radial points on the h2 section. evolution of the temperature and hydrogen storage fields is spatially manifested as an annular reaction propagation mode and non-uniform distribution characteristics.

The temperature gradients and storage capacity discrepancies arising from the aforementioned temporal evolution are further manifested at the spatial scale as pronounced axial–radial non-uniformity. Throughout the hydrogen absorption process, the temperature and hydrogen storage fields exhibit distinct spatial heterogeneity. Along the axial direction, the temperature field conforms to a distribution pattern of high in the middle and low at both ends, whereas the hydrogen storage capacity displays the opposite trend, specifically characterized as low in the middle and high at both ends. In the radial direction, the corresponding distribution patterns are featured by low temperature at the wall and high temperature at the center and rapid adsorption at the wall and slow adsorption at the center, as illustrated in Figure 7a,b. Analogous characteristics pertaining to the spatial non-uniformity of thermal fields and reaction behaviors have been documented in the work of Hasnain [19]. The underlying physical mechanism stems from the spatial mismatch between three key factors: reaction heat accumulation, thermal diffusion pathways, and gas-phase hydrogen replenishment capacity. Specifically, the heat released during the exothermic hydrogen absorption reaction tends to accumulate locally, giving rise to elevated temperatures that increase the equilibrium hydrogen pressure and thereby impede the reaction kinetics. Concurrently, the inherently low thermal conductivity of the alloy matrix, combined with the elongated diffusion pathways within the hydride bed, hinders the efficient dissipation of accumulated heat. Moreover, the migration of hydrogen molecules into the inner layers of the alloy is constrained by the extended transport distance and the associated increase in permeation resistance. This thermally driven self-inhibition mechanism, in conjunction with the limitations of gas-phase mass transport, induces a pronounced kinetic lag in the core region of the hydride bed—a phenomenon that is further corroborated by findings from recent review articles [41].

Axially, the bottom-adjacent segment h1 exhibits the fastest reaction rate, with a peak temperature rise of merely 13 K. This behavior can be ascribed to its close proximity to the cooling wall, which affords a shortened pathway for solid-phase heat conduction and thus facilitates efficient thermal dissipation. By contrast, the middle segment h2 of the hydride bed is situated in a region where both axial and radial hydrogen replenishment are substantially constrained: it lacks direct and sufficient gas-phase hydrogen supply from the top, while also featuring the longest average heat transfer distance to the outer cooling wall. The resultant severe heat accumulation elevates the peak temperature of this segment to 327 K; such pronounced thermal buildup induces strong thermodynamic inhibition, leading to a minimum hydrogen storage capacity of only 1.21 wt% and thereby embodying the typical characteristics of synergistic heat and mass transport limitation. The segment h3, adjacent to the hydrogen inlet, benefits from direct and continuous contact with the incoming hydrogen flow, which ensures the amplest gas-phase replenishment. Correspondingly, this segment displays the highest initial hydrogen storage rate and a rapid temperature surge. Nevertheless, the convective cooling effect of the low-temperature inlet hydrogen suppresses its peak temperature, which remains 9 K lower than that of the h2 segment. Within the reserved void space of the reactor, the temperature variation in the h4 segment conforms to the overall thermal evolution trend of the hydride bed, whereas the topmost h5 segment maintains a relatively stable temperature profile, as it is only subject to weak convective effects (Figure 7c,d). Radially, the hydrogen storage capacity at all positions jumps to ~0.175 wt% in a short time, as the local equilibrium pressure inside the bed is far lower than the external supply pressure. Subsequently, distinct differences emerge rapidly, and radial heterogeneity intensifies. Position R6 near the wall experiences a minimal 4 K temperature rise that recovers quickly due to wall cooling. Moving toward the center, positions R5 to R3 show continuous declines in temperature rise and storage rate, a consequence of aggravated heat accumulation and more restrictive gas replenishment. However, toward the reaction end, non-monotonic fluctuations and a storage rate rebound are observed at central positions R1–R3 (Figure 7e–g). This behavior stems from enhanced axial cooling, the gradual clearing of gas replenishment channels, and the centripetal contraction of the annular reaction front. Physically, as the reaction in the outer annular region concludes, heat generation stops, allowing core heat to dissipate outward. Meanwhile, unobstructed gas pathways mitigate the thermal self-inhibition effect in the center, enabling the reaction front to complete centripetal contraction. This phenomenon reflects the spatial shift in reaction control from kinetic dominance to thermal-diffusion dominance, consistent with recent review findings [42]. Clearly, the axial–radial spatial heterogeneity during sorption is not caused by initial condition variations, but is an inevitable result of the spatiotemporal coupling mismatch between reaction thermal effects, thermal diffusion capacity, and gas replenishment pathways.

### 3.2. Heat-Mass Transfer Characteristics During H_2_ Desorption

Unlike the hydrogen absorption process, which relies on exothermic heat generation to trigger temperature rises, the hydrogen desorption process is fundamentally a strongly endothermic process limited by the rate of heat compensation. Its kinetic behavior is profoundly governed by the spatiotemporal mismatch between the endothermic desorption rate, the external heat supply, and the internal thermal diffusion capacity of the bed. The rapid decrease in bed temperature causes a significant drop in local equilibrium pressure, which weakens the thermodynamic driving force for desorption and exerts a typical, sustained thermally induced self-inhibition effect on the reaction. Simultaneously, the low thermal conductivity of the bed and the progressively lengthening heat conduction pathways restrict the transfer of heat toward the core region, leading to pronounced spatial heterogeneity in the endothermic reaction front. From the perspective of thermal–mass coupling imbalance, this section systematically analyzes the temporal evolution and spatial distribution characteristics of the temperature and hydrogen storage fields during desorption. The objective is to elucidate the spatiotemporal dynamic response mechanisms of the desorption reaction dominated by multi-physical fields competition.

**Figure 8:**
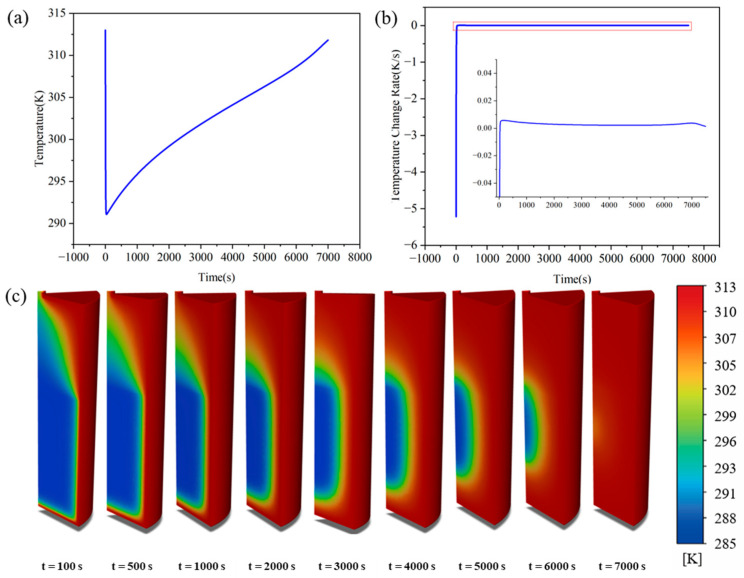
Temperature evolution during the hydrogen desorption process: (**a**) average bed temperature; (**b**) temperature change rate; (**c**) temperature distribution at different moments.

The temporal evolution of the temperature field during desorption directly reflects the dynamic competition among external heat supply, bed thermal diffusion, and the endothermic reaction. The temperature evolution exhibits three typical stages, which are the rapid cooling stage, the non-linear heat compensation recovery stage, and the slow thermal diffusion equilibrium stage, as illustrated in Figure 8a,b. Such multi-stage temperature evolution behaviors have been systematically reported in studies of MHB desorption [29] and are generally attributed to the dynamic imbalance between the endothermic reaction rate and the capacities of external heat supply and thermal diffusion.

The stage partitioning of the desorption process also adopts quantitative criteria based on the temperature change rate, but considering that the overall reaction rate and thermal response magnitude of the desorption process are lower than those of the absorption process, the criterion thresholds have been adjusted accordingly. The transition point from Stage I to Stage II is defined as the moment when the characteristic temperature change rate of the bed approaches zero (dT/dt≈0), corresponding to the instantaneous balance between the reaction endothermic rate and the external heat supply rate. The transition from Stage II to Stage III is defined as the moment the system enters the low-speed thermal response interval; when ∣dT/dt∣ drops below the threshold of $10^{-3}\;K/s$, it is considered that both the temperature field and the reaction rate have entered a slow-changing stage. The stage division for the hydrogen release capacity and reaction rate follows the same time boundaries as the temperature characteristic nodes to ensure the consistency of the multi-physical quantity analysis.

The initial stage (t < 100 s) is the rapid cooling phase, which is entirely controlled by the strong endothermic nature of the reaction. The bed temperature drops sharply within seconds, with the cooling rate peaking at approximately 5.5 K/s. Due to the low thermal conductivity of LaNi_5_, the external heat compensation rate is far lower than the internal reaction heat absorption rate. Consequently, the bed is forced to release its sensible heat to provide the desorption enthalpy required for the reaction, leading to a transient sharp temperature drop. Early studies have noted that this rapid temperature drop is a typical thermal response during metal hydride desorption. Its essence lies in the dynamic imbalance between reaction heat absorption and bed heat compensation capacity [43], which induces a thermally induced self-inhibition effect and directly leads to the subsequent decay of the reaction rate. The middle stage (100 < t < 2000 s) is the non-linear heat compensation recovery phase, representing a period of dynamic competition between the endothermic reaction and external heat supply. After reaching a minimum of 291 K, the temperature undergoes a brief rapid rebound, followed by a gradual decrease in the heating rate. The heating rate declines from 0.05 K/s to 0.002 K/s, entering a distinct non-linear recovery process. At this point, the continuous external heat supply exceeds the endothermic demand of this stage. The brief rapid temperature rebound indicates that the reaction heat absorption rate has significantly decayed due to thermally induced self-inhibition and has fallen below the external heat compensation rate. As solid-phase conduction and limited gas-phase convection gradually replenish heat to the interior of the bed, the temperature recovery re-elevates the local equilibrium pressure. This alleviates the reaction rate inhibition caused by the initial low temperatures and establishes a dynamic coupling between reaction heat absorption and heat supply from the environment and the bed body, resulting in non-monotone temperature evolution. The late stage (t > 2000 s) is the phase dominated by slow thermal diffusion. At this time, the reaction rate drops to approximately 0.001 K/s and the desorption heat absorption attenuates significantly. The temperature variation is entirely governed by the macro-thermal diffusion resistance within the bed and limited compensation from gas-phase convection. Classical transient model studies indicate that after the reaction driving force continues to weaken, the bed temperature recovery process is typically dominated by thermal diffusion capacity, exhibiting characteristic diffusion-controlled features [44,45]. As shown at t = 3000 s and subsequent points in Figure 8c, the internal temperature field recovers slowly and spatial differences remain significant, which directly leads to the kinetic long-term tailing phenomenon in the subsequent evolution of hydrogen storage capacity.

**Figure 9:**
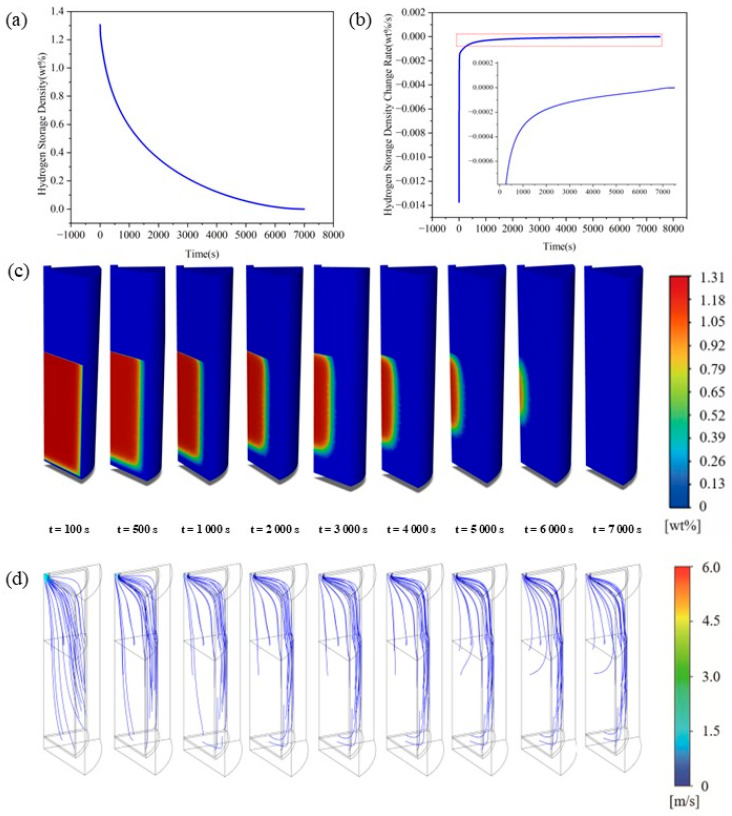
Hydrogen storage capacity and hydrogen flow field during the desorption process: (**a**) average bed hydrogen storage capacity; (**b**) hydrogen storage rate; (**c**) hydrogen storage distribution at different moments; (**d**) hydrogen flow field distribution at different moments.

The temporal variation of hydrogen storage capacity serves as a direct characterization of desorption kinetics. Its evolution is jointly constrained by the endothermic reaction characteristics, the heat compensation capacity of the bed, and the hydrogen diffusion resistance. The intense coupling between reaction kinetics and multi-physical field transport characteristics during desorption results in a pronounced non-linear evolution of the hydrogen storage capacity over time. Based on the study by Mayer et al. [44] and the numerical simulation results of this model, the temporal evolution of hydrogen storage capacity during desorption is categorized into three representative stages: the rapid desorption stage, the heat-compensation-limited desorption front inward propagation stage, and the diffusion-limited reaction attenuation stage, as shown in Figure 9a,b.

The initial stage (t < 100 s) is the rapid desorption phase, where the storage capacity decreases sharply at a rate exceeding 0.001 wt%/s, driven by the maximum mass transfer driving force. During this stage, the solid-phase heat conduction pathways near the wall are short, allowing for rapid temperature recovery. Consequently, the local temperature near the wall is higher than in the interior, leading to a higher local equilibrium pressure. Moreover, the hydrogen diffusion resistance in the vicinity of the wall is at its minimum, enabling hydrogen to be rapidly discharged through low-resistance axial–radial channels. An effective partial pressure difference has not yet developed within the reactor interior. As the reaction proceeds, the bed temperature and equilibrium pressure decrease, weakening the reaction driving force and significantly reducing the hydrogen release rate per unit time. This trend is consistent with the reaction-dominated desorption at the onset of the process reported in the literature [41].

The middle stage (100 < t < 2000 s) is the heat-compensation-limited desorption front inward propagation phase. The storage capacity decay rate continuously attenuates from 0.001 wt%/s to approximately 0.0002 wt%/s, although substantial desorption still occurs. The drastic decrease in core temperature leads to a sharp drop in local equilibrium pressure, making the desorption driving force limited by the external heat replenishment rate. This process is described as the inward propagation of a thermally limited desorption front to emphasize its nature of being controlled by thermal diffusion. During this stage, despite the decline in the overall reaction rate, a certain partial pressure difference and relatively short solid-phase diffusion distances are maintained internally. This allows the desorption process to sustain a moderate rate, as indicated by the curves for this period in Figure 9a,b.

**Figure 10:**
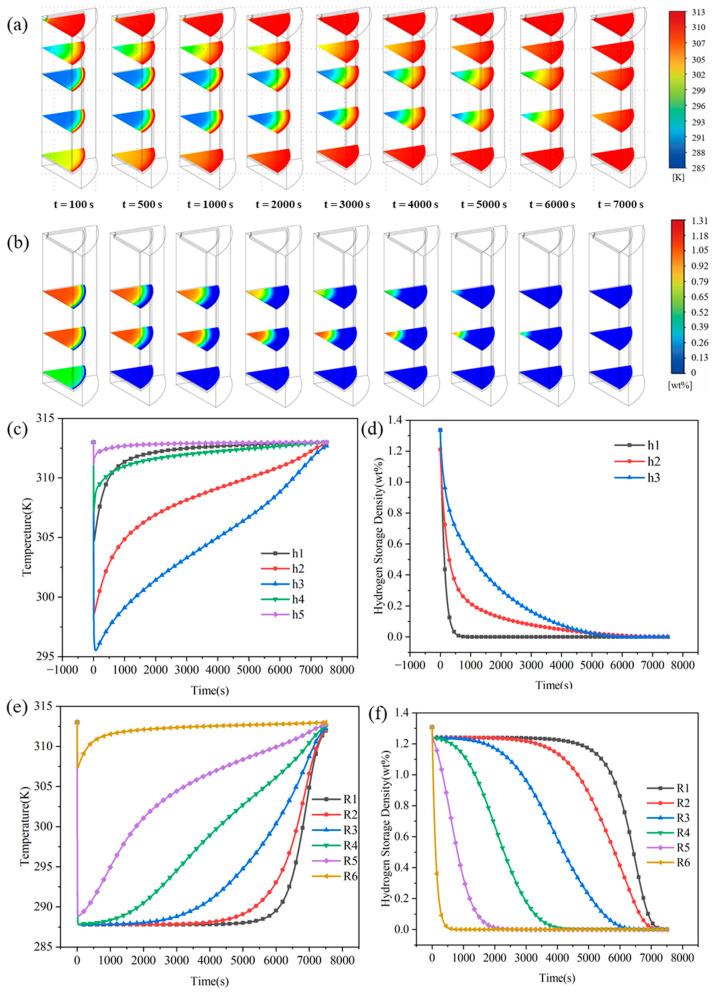

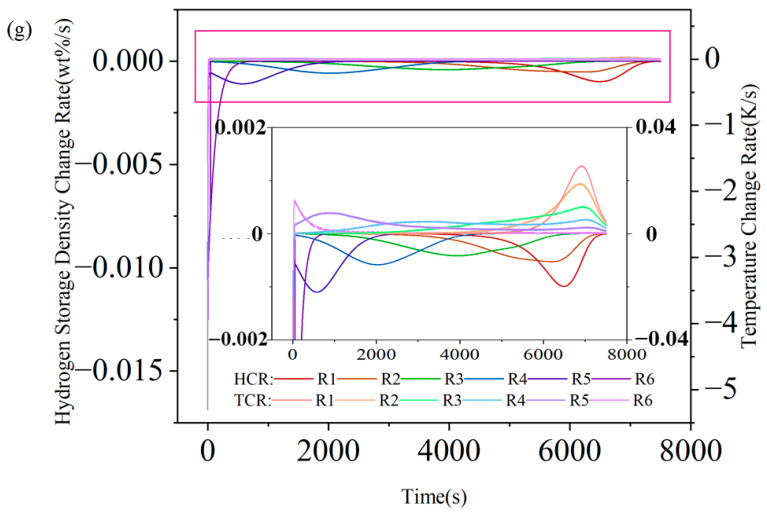
Axial and radial spatial evolution during desorption: (**a**) axial temperature distribution; (**b**) axial hydrogen storage capacity distribution; (**c**,**d**) temperature and hydrogen storage capacity at different cross-sections; (**e**–**g**) temperature, hydrogen storage capacity, and their rates of change at various radial points on the h2 section.

The late stage (t > 2000 s) is the diffusion-limited reaction attenuation phase, where the storage capacity curve flattens and the desorption rate approaches zero. At this point, the outer regions have basically completed the reaction, and the deeper particles face dual resistances. From a microscopic perspective, the solid-phase diffusion resistance of residual hydrogen atoms within the metal lattice or hydride layer increases significantly [46]. Macroscopically, hydrogen released from deeper layers must traverse the longest pathways to escape. The high permeation resistance along the path combined with an extremely weak pressure driving force ultimately makes the reaction limited by mass transfer efficiency. This stage demonstrates the synergistic inhibition of multi-scale diffusion resistance on the kinetic performance during the late phase of desorption. The asynchronous temporal evolution of the temperature field and hydrogen storage capacity further manifests as significant spatial heterogeneity during the desorption process. Axially, the reactor exhibits a “high temperature at the bottom, low temperature at the top” profile, which corresponds to a “fast storage decay at the bottom, slow decay at the top” pattern. Radially, the system is characterized by “high temperature at the wall, low temperature at the center” and “fast desorption at the wall, slow desorption at the center,” as illustrated in Figure 10a,b. This spatial heterogeneity is jointly driven by the significant cooling of the inner layers caused by endothermic desorption, the limited external heat replenishment, and the transport characteristics of preferential hydrogen discharge along the wall. These factors culminate in a complex distribution pattern influenced by thermal–mass coupling. In the axial dimension, the h1 section adjacent to the reactor bottom is in direct contact with the wall, allowing for rapid heat compensation through a short solid-phase conduction pathway. Simultaneously, the desorbed hydrogen escapes through low-resistance channels near the wall, resulting in a minimum temperature drop of only 9 K, the fastest temperature recovery, and the highest storage decay rate. The h2 section receives heat compensation from the wall but is also subjected to the convective cooling of low-temperature hydrogen flowing from bottom to top. Consequently, its temperature drop and desorption rate fall between those of the h1 and h3 sections, exhibiting a typical axial transition characteristic. The top section h3 is located near the outlet, where desorbed hydrogen flowing upward arrives last. This area experiences the maximum cumulative convective cooling effect, leading to a maximum temperature drop of 18 K and the slowest recovery. This delayed recovery is consistent with the findings of Muthukumar et al. [45], who attributed this phenomenon to the cumulative cooling effect of the desorbed hydrogen flow. Within the reserved expansion space, the h4 section is directly affected by the escaping cold hydrogen, showing significant and prolonged temperature variations. In contrast, the h5 section is only briefly cooled by the passing hydrogen and rapidly recovers to its initial temperature, as shown in Figure 10c,d.

In the radial dimension, all positions rapidly desorb to approximately 1.24 wt% during the initial stage because the outlet pressure is significantly lower than the bed equilibrium pressure. Subsequently, the desorption behavior gradually exhibits a distinct bifurcation. Point R6 adjacent to the outer wall receives sustained external heat compensation, resulting in a minimum temperature drop of only 6 K and the fastest decay in hydrogen storage capacity. This forms a characteristic preferential desorption of the outer layers. Such steep radial gradients highlight the severe inherent heat transfer limitations of metal hydride beds, a phenomenon previously reported in the experimental studies of Mayer et al. [44] and Askri et al. [40]. Moving centripetally toward positions R5 to R3, the rates of temperature recovery and storage capacity decay gradually slow down due to the progressive attenuation of heat compensation and the extension of hydrogen escape pathways. Notably, non-monotonic fluctuations in the desorption rate are observed at points R1 to R2 near the core during the late stage of the reaction. These fluctuations are attributed to the gradual decrease in the axial temperature gradient during the mid-to-late phase, which allows heat from the bottom and wall regions to conduct toward the center. As the reaction in the outer layers completes, the surrounding regions no longer compete for heat compensation or hydrogen discharge channels, facilitating the formation of a locally favorable partial pressure gradient in the core. Additionally, cold hydrogen flowing radially toward the wall exerts a stepwise cooling effect on points along its path, as illustrated in Figure 10e–g. These complex fluctuation characteristics, which are typically smoothed over in simplified 2D models [21], are accurately captured by the present 3D model, providing deeper insights into thermal–mass coupling phenomena. In summary, under engineering-scale conditions, the hydrogen desorption process exhibits staged evolution characteristics dominated by the heat input capacity. Its temporal kinetics and spatial heterogeneity fundamentally originate from the coupling mismatch among the endothermic reaction, thermal diffusion, and the hydrogen release process. This stands in sharp contrast to the kinetic behavior driven by reaction exothermicity during the absorption process.

### 3.3. Heat-Mass Transfer Characteristics Among Different Heat-Exchange Structures

To systematically elucidate the influence mechanisms of internal heat-exchange structures on the coupled thermal–mass transfer performance of MH reactors, three representative geometric configurations are selected for comparative analysis, which are the straight pipe structure, spiral tube structure, and honeycomb structure. These three structures are kept consistent in terms of total dimensions, material loading, and operating conditions. This approach aims to eliminate the interference of scaling effects and filling variations on transfer behavior, thereby establishing the geometric configuration as the dominant independent variable. Integrating numerical simulation results with thermal resistance network and seepage theories, this section constructs a mechanistic framework to understand how geometric configurations synergistically enhance thermal–mass transfer. This framework is analyzed across three levels, specifically the expansion of heat-exchange area, the reconstruction of hydrogen flow pathways, and the spatial distribution of reaction heat sources. The research methodology responds to the evolutionary trend discussed by Miao et al. [37], which advocates for a shift in MH reactor thermal management from simple structural reinforcement toward mechanism-driven design.

**Figure 11:**
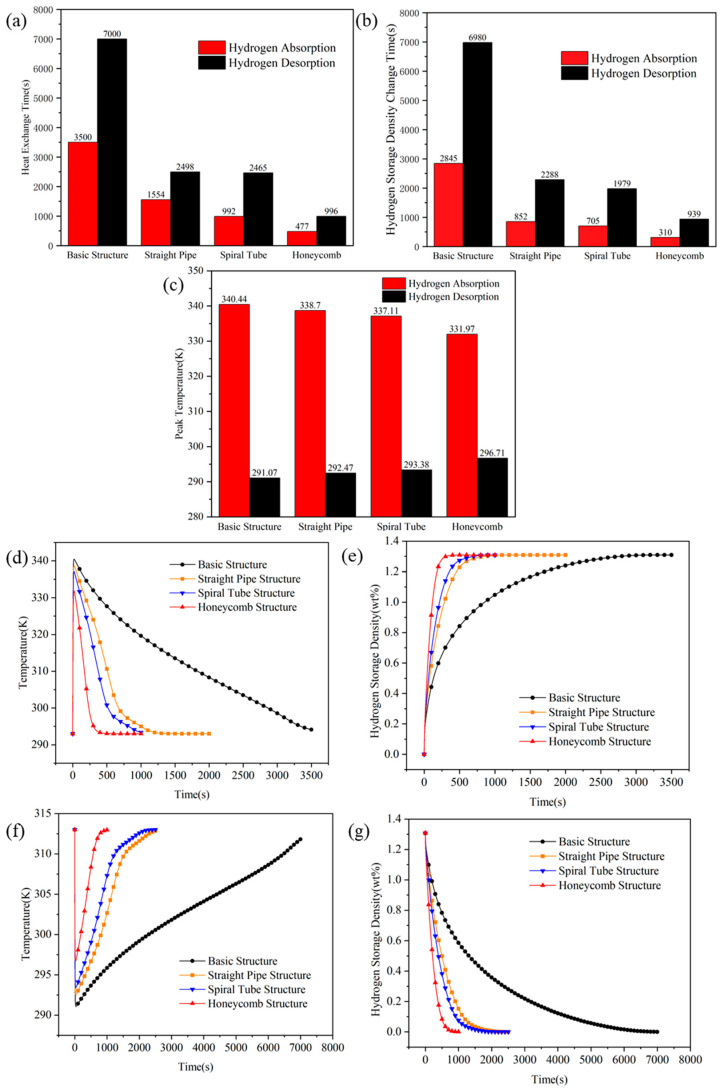
Comparison of hydrogen adsorption and desorption performance between the basic structure and various internal heat-exchange structures: (**a**) heat-transfer completion time; (**b**) reaction completion time; (**c**) peak temperature during sorption; (**d**,**e**) temperature and hydrogen storage capacity during absorption; (**f**,**g**) temperature and hydrogen storage capacity during desorption.

From the perspective of heat transfer, the internal structures significantly reduce the overall thermal resistance of the bed by maximizing the solid–fluid interface area and minimizing the characteristic thermal diffusion length. Numerical results indicate that the heat dissipation duration for the basic structure during absorption exceeds 3500 s, whereas this is shortened to 1554 s (~55.6%), 992 s (~71.7%), and 477 s (~86.4%) for the straight pipe, spiral tube, and honeycomb structures, respectively. During the desorption stage, the heat compensation duration for the basic structure exceeds 7000 s, while it is reduced to 2498 s (~64.3%), 2465 s (~64.8%), and 996 s (~85.8%) for the straight pipe, spiral tube, and honeycomb structures, respectively. This stepwise performance improvement matches the theoretical analysis by Mou et al. [16] concerning the effective thermal conductivity of LaNi_5_ hydride beds, which suggests that heat conduction in low-conductivity matrices is primarily restricted by solid-phase contact thermal resistance. The honeycomb structure substantially compresses the maximum heat transfer path from 25 mm in the basic structure to 3 mm, effectively constructing a high-density thermal conductive skeleton. Detailed comparisons of the heat transfer completion times, reaction kinetics, and peak temperature variations for all investigated architectures are summarized in Figure 11.

Bed heat transfer control mechanism from macroscale bulk conduction limitation to local thermal equilibrium control characterized at the unit scale. Consequently, the original large-scale heat transfer problem is decomposed into microscale local thermal equilibrium issues. The peak temperature difference during sorption is sequentially reduced from 49.37 K for the basic structure to 46.23 K, 43.73 K, and 35.26 K for the straight pipe, spiral tube, and honeycomb structures, respectively. These results indicate that the heat source distribution has been effectively reconfigured. This corroborates the perspective of Bai et al. [23] in their study on fin optimization, specifically that increasing the heat-exchange surface area not only enhances the reaction rate but also improves the spatial uniformity of reaction kinetics by suppressing localized hot spots.

From the perspective of mass transfer, internal structures function not only as heat-transfer fins but also as gas flow channels. By increasing the effective seepage boundaries, these structures reduce the permeation resistance of hydrogen within the porous medium, thereby enhancing the overall reaction rates. Simulation results demonstrate that during absorption, the incorporation of straight pipe, spiral tube, and honeycomb structures shortens the completion time from 2845 s (basic structure) to 852 s, 705 s, and 310 s, respectively, corresponding to reduction rates of 70.1%, 75.2%, and 89.1%. For the desorption process, the duration is reduced from 6980 s for the Basic structure to 2280 s, 1979 s, and 939 s for the three optimized structures, with reduction rates reaching 67.3%, 71.7%, and 86.6%, respectively. These findings mechanistically corroborate the multi-field coupling resistance map theory proposed by Wang et al. [8], which suggests that the gas-phase pressure drop can become a critical bottleneck for reaction front propagation during intense reaction phases. While straight pipes and spiral tubes provide continuous low-resistance axial pathways, the honeycomb structure further partitions the bed into numerous independent micro-reaction units, enabling hydrogen to reach reaction sites via the shortest possible distance. Notably, the maximum reduction rate of 86.6% during desorption validates the viewpoint of Kudiiarov et al. [7]. Specifically, optimizing heat-exchange structures can simultaneously improve the gas flow network, effectively alleviating the kinetic tailing phenomenon caused by the combined effects of pressure gradient decay and extended diffusion pathways during the late reaction stages. This phenomenon is widely observed in the desorption processes of low-thermal-conductivity metal hydride beds.

To further elucidate the disparities in heat and mass transfer performance among the different structures, it is essential to analyze the regulatory mechanisms of thermal diffusion, gas transport pathways, and the distribution of reaction heat sources. The most direct mechanism for enhanced thermal diffusion within internal structures is the stepped expansion of the effective heat-exchange area and the introduction of a continuous thermal conductive skeleton. Compared with the basic structure, the heat-exchange areas of the straight pipe, spiral tube, and honeycomb structures increase by 49.9%, 85.4%, and 669%, respectively, corresponding to area increments of 4701.6 mm^2^, 8043.6 mm^2^, and 63,030 mm^2^. The thermal conductivity of the 316L stainless steel used for the internal structures is 6.7 times that of the LaNi_5_ alloy, which not only improves the effective thermal conductivity of the bed but also fundamentally alters the heat transfer mode. Specifically, the heat transfer shifts from the long-distance radial conduction dominated by a single outer wall in the basic structure to short-distance local thermal equilibrium governed by the internal distributed metal skeleton. Notably, although the heat-exchange area of the spiral tube structure is further expanded compared to the straight pipe structure, simulation results indicate that the magnitude of performance improvement is limited. This observation is consistent with recent comparative studies between straight and helical tubes by Mou et al. [17], suggesting that an increase in surface area within a hydride bed does not necessarily translate linearly into an enhancement of the effective thermal conductivity. The present study further demonstrates that a substantial breakthrough in the heat transfer bottleneck, which is dominated by contact thermal resistance, can only be achieved when the highly conductive skeleton forms a high-density and short-scale network in space. In contrast, the honeycomb structure significantly increases the specific surface area per unit volume while effectively overcoming the constraints of contact thermal resistance. This finding is highly self-consistent with the observed improvements in temperature field uniformity and the significant reduction in peak temperature differences.

Secondly, the internal structures significantly enhance the equivalent permeability of the bed by reconstructing hydrogen flow pathways and shortening mass transfer distances. Quantitatively, the straight pipe structure, spiral tube structure, and honeycomb structure reduce the maximum mass transfer distance by 50%, 50%, and 88%, respectively. These reductions drive a decrease in absorption completion time by 70.1–89.1% and desorption completion time by 67.3–86.6%. This flow field reconfiguration effect aligns with the theoretical predictions of Hasnain et al. [19] regarding the impact of reactor geometric parameters, which state that the geometric configuration directly determines the resistance network of the gas-phase flow. Specifically, the straight pipe structure establishes low-resistance axial diffusion pathways throughout the bed, accelerating axial gas transport. The spiral tube structure utilizes its geometric curvature to induce axial–radial secondary flows. As identified by Wu et al. [26], this secondary flow mechanism effectively disrupts the boundary layer, providing additional replenishment channels in the radial direction. In contrast, the honeycomb structure adopts a discretization strategy by partitioning the bed into 61 independent micro-fluidic units. This configuration forms a multi-channel parallel transport network that minimizes the restrictions imposed by local pressure gradients on the reaction rate. It should be emphasized that this mass transfer enhancement is not merely an indirect consequence of temperature field improvements but originates from the direct reshaping of the gas-phase resistance network by the geometric configuration. Consequently, the hydrogen transport mechanism transitions from being solely porous-medium-dominated to a parallel dominance of both the porous medium and explicit channels.

**Figure 12:**
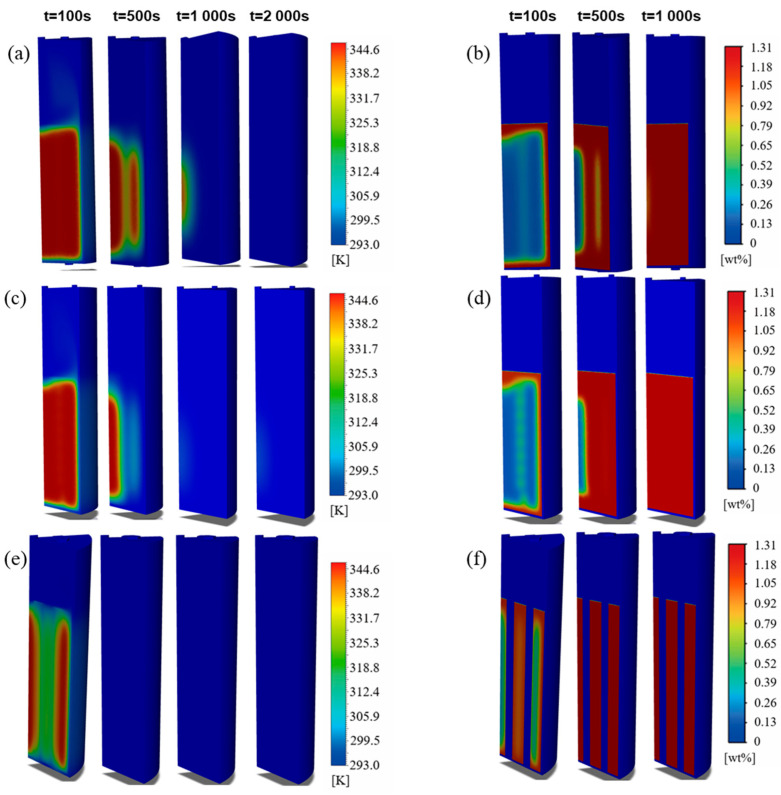
Spatial distributions of temperature field and hydrogen storage capacity for different internal structures during absorption: (**a**,**b**) straight pipe structure; (**c**,**d**) spiral tube structure; (**e**,**f**) honeycomb structure.

**Figure 13:**
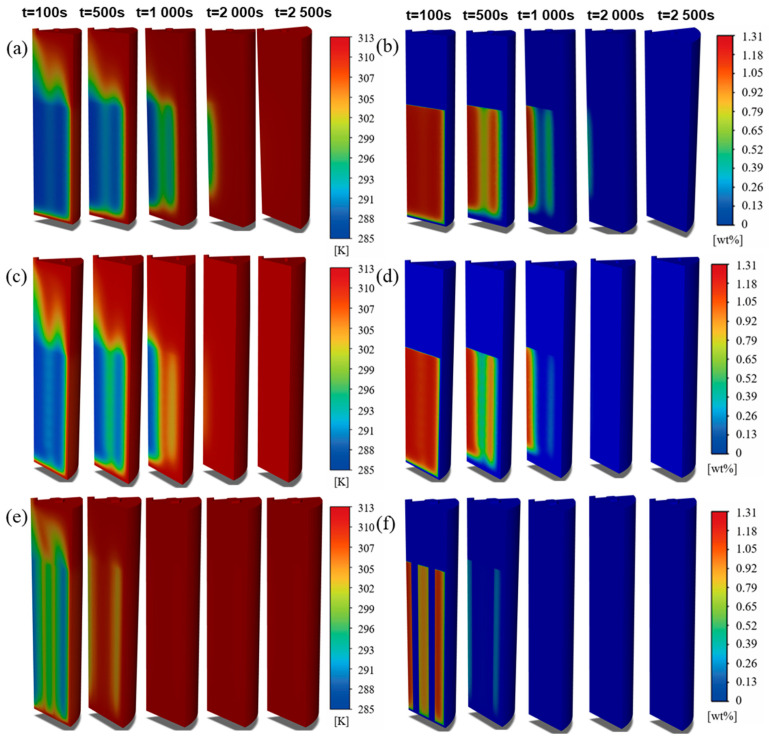
Spatial distributions of temperature field and hydrogen storage capacity for different internal structures during desorption: (**a**,**b**) straight pipe structure; (**c**,**d**) spiral tube structure; (**e**,**f**) honeycomb structure.

Finally, the internal structures achieve deep-level coupling optimization of heat and mass transfer by synergistically regulating the spatial distribution of reaction heat sources. The peak temperature difference during sorption is sequentially reduced from 49.37 K in the Basic structure to 35.26 K in the honeycomb structure. Given that the equilibrium pressure of LaNi_5_ follows the Van’t Hoff equation and is highly sensitive to temperature, a reduction in temperature gradients directly implies a decrease in local equilibrium pressure differences within the bed. This consequently leads to a more uniform spatial distribution of the thermodynamic driving force. As shown in the spatiotemporal evolution in Figure 12 and Figure 13, the straight pipe structure redistributes heat sources into strip-like patterns, thereby breaking the limitation of layer-by-layer inward propagation observed in the Basic structure (see Figure 12 and Figure 13a at t = 100 s and 500 s). The spiral tube structure further attenuates 2D temperature gradients, causing the heat source distribution to deviate from a regular annular pattern as the reaction proceeds and leading to enhanced internal reaction uniformity. In contrast, the honeycomb structure highly discretizes the heat sources in space, which facilitates the synchronous propagation of the reaction front in both axial and radial directions (see Figure 12 and Figure 13e,f at t = 100 s). Therefore, the performance advantages of the honeycomb structure do not stem from a simple superposition of individual heat or mass transfer mechanisms but originate from a systematic reconfiguration of the spatiotemporal distribution of reaction heat sources. This reconfiguration constitutes the fundamental reason for its ability to achieve cross-scale synergistic reinforcement in hydrogen sorption kinetics. In summary, internal heat-exchange structures achieve a spatiotemporal reconfiguration of the thermal–mass coupling imbalance by synergistically regulating the heat-transfer area, gas transport pathways, and heat source distribution. Among these, the honeycomb structure exhibits the greatest potential for comprehensive enhancement due to its superior performance in minimizing transport resistance and maximizing reaction uniformity.

## 4. Conclusions

Both hydrogen absorption and desorption processes exhibit a three-stage evolution, shifting from reaction kinetics dominance to transfer limitation. This behavior stems from the nonlinear spatiotemporal scale mismatch between the intensity of reaction heat release/absorption and the bed’s comprehensive thermal–mass transfer capacity. A distinct thermal self-inhibition effect occurs during absorption, while desorption is constrained by the simultaneous decay of heat compensation and hydrogen diffusion, resulting in a characteristic long-tail kinetic response.

Under the dual constraints of low effective thermal conductivity and mass transfer resistance, the reaction front forms a stable “inverse core-surface distribution pattern,” which is the primary source of spatial heterogeneity. Driven by the dynamic imbalance between local heat transfer intensity and long-range mass transfer rates, this distribution is continuously amplified via the equilibrium pressure feedback mechanism, constituting the key limiting mechanism at the reactor scale.

The core function of internal structural enhancement lies in the synergistic reconfiguration of the thermal–mass resistance network rather than simple heat transfer augmentation. Under constant mass loading, the honeycomb architecture, with its parallel micro-unit conductive skeleton, significantly expands the heat exchange area (by ~669%) while simultaneously reconstructing gas transport pathways and shortening characteristic mass transfer distances. This dual compression of resistance and spatial redistribution of heat sources reduced the peak temperature difference from 49.37 K to 35.26 K and shortened absorption/desorption times by 89.1% and 86.6%, respectively. These multi-indicator outcomes establish a rigorous quantitative evidence chain for structural decoupling.

Based on these mechanistic insights, this study establishes a mechanism-driven design framework for structural decoupling to resolve thermal–mass coupling mismatch. By compressing characteristic length scales and constructing a distributed transfer-transport network, synergistic control of multi-physical field dynamic responses is achieved. This methodology is applicable to other hydride systems with low conductivity and high reaction heat. It should be noted that the current model assumes constant porosity and fixed volume, without explicitly coupling the dynamic feedback of particle expansion on pore structures and contact resistance, and the boundary conditions are primarily used for relative performance comparisons. Future work will incorporate the time-varying effects of bed structural parameters to extend the applicability of the model.

## Figures and Tables

**Table 1:** Comprehensive geometric dimensions and boundary conditions of the reactors.

Category	Parameter	Symbol	Unit	Value
Reactor Body	Canister height	*H*	mm	102
	Canister radius	*R*	mm	26
	Wall thickness	*L*	mm	1
	H_2_ inlet/outlet diameter	*D*	mm	4
Hydride Bed	Bed filling height	*h*	mm	60
	Bed filling radius	*r*	mm	25
Straight Pipe	Number of channels	*n*	-	6
	Outer/inner diameter	*OD*/*ID*	mm	4/3
Spiral Tube	Number of channels	*n*	-	6
	Helix pitch/number of coils	*p*/*N*	mm	6/14
	Helix radius	*R_hel_*	mm	2.5
	Outer/inner diameter	*OD*/*ID*	mm	2.5/1.5
Honeycomb	Cell-inscribed circle diameter	*d_cell_*	mm	6
	Cell wall thickness	*L_cell_*	mm	1
	Pipe-inscribed circle diameter	*d_pipe_*	mm	6
Boundary	HTF (water) inlet velocity	*v*	m/s	1
	Initial bed temperature	*T*_0_	*K*	293
	Constant inlet pressure	*P_in_*/*P_out_*	MPa	1/0.1

**Table 2:** Various thermo-physical parameters of the metal hydride and their values used in the present work [27,28].

Name	Symbol	Value	Unit
Activation energy for absorption reaction	*E_a_*	21,179.6	J/mol
Activation energy for desorption reaction	*E_d_*	16,473	J/mol
Absorption rate constant	*C_a_*	59.187	1/s
Desorption rate constant	*C_d_*	9.57	1/s
Effective heat convection coefficient	*h*	1652	W/(m^2^·K)
Permeability	*Κ*	10^−8^	m^2^
Effective thermal conductivity of the bed	*λ_eff_*	2.4	W/(m·K)
Porosity of bed	*ε*	0.5	
Enthalpy of formation	Δ*H*	1.539 × 10^7^	J/kg
Saturated metal hydride density	*ρ_sat_*	7259	kg/m^3^
Hydrogen-free metal hydride density	*ρ_emp_*	7164	kg/m^3^
Specific heat of the metal	*C_p,s_*	419	J/(kg·K)
Molecular mass of hydrogen	*M_g_*	2.0159	kg/kmol
Specific heat of hydrogen gas	*C_p,g_*	14,890	J/(kg·K)
Weight fraction of maximum	wt%	1.307%	
Universal gas constant	*R*	8.314	J/(mol·K)

**Table 3:** Filling height of metal hydride alloy in different internal structural configurations.

Structure	Basic Structure	Straight Pipe	Spiral Tube	Honeycomb
Material filling height	60 mm	62.35 mm	60.85 mm	74.56 mm

**Table 4:** Selected heights of different cross-sections within various internal structural configurations.

Structure	h1	h2	h3	h4	h5
Basic Structure	0	30	60	80	100
Straight Pipe	0	31.175	62.35	81.175	100
Spiral Tube	0	30.425	60.85	80.425	100
Honeycomb	0	37.28	74.56	87.28	100

**Table 5:** Parameter values used in the mass source term.

Reaction Type	C	E	$P_{\mathrm{eq}}$	$\rho^{*}$
Hydrogen absorption ($P_{g}>P_{\mathrm{eq},a}$)	$C_{a}$	$E_{a}$	$P_{\mathrm{eq}\_a}$	$\rho_{\mathrm{sat}}$
Hydrogen desorption ($P_{g}<P_{\mathrm{eq},d}$)	$C_{d}$	$E_{d}$	$P_{\mathrm{eq}\_d}$	$\rho_{\mathrm{emp}}$

**Table 6:** Parameter values used in the Van’t Hoff equation.

Reaction Type	A	B
Hydrogen absorption	10.7	3704.6
Hydrogen desorption	10.57	3704.6

**Table 7:** Comparison of boundary conditions and thermophysical properties between the present work and the reference work [27].

Parameter	Present Work	Reference Work
*T*_0_ (K)	293.15	293.15
*T_ev_* (K)	293.15	293.15
*h* (W/m^2^·K)	2.4	2.4
*P*_in_ (MPa)	0.8	0.8
*λ*_eff_ (*W*/(m·K)	2.4	2.4
*ε*	0.5	0.5
Validation point location	(15, 35)	Point A
*ρ_emp_* (kg/m^3^)	7164	7164

## Data Availability

The original contributions presented in this study are included in the article. Further inquiries can be directed to the corresponding authors.
